# Interchangeability between the Data Obtained by Two Powermeters during Road Cycling Competitions: A Case Study

**DOI:** 10.3390/ijerph192416446

**Published:** 2022-12-08

**Authors:** Javier Iglesias-Pino, Alba Herrero-Molleda, Jaime Fernández-Fernández, Juan García-López

**Affiliations:** Faculty of Physical Activity and Sports Sciences, AMRED, Human Movement and Sports Performance Analysis, Universidad de León, 24071 León, Spain

**Keywords:** cycling, competition analysis, power profile, critical power

## Abstract

Various power meters are used to assess road-cycling performance in training and competition, but no previous study has analyzed their interchangeability in these conditions. Therefore, the purpose was to compare the data obtained from two different power meters (PowerTap vs. Power2Max) during cycling road races. A national-level under-23 male competitive cyclist completed six road-cycling official competitions (five road races and one individual time trial), in which power output was simultaneously registered with the two power meters. After this, the main power output variables were analyzed with the same software. The average and critical power obtained from the PowerTap power meter were slightly lower than from the Power2Max power meter (3.56 ± 0.68 and 3.62 ± 0.74 W·kg^−1^, 5.06 and 5.11 W·kg^−1^, respectively), and the correlations between both devices were very high (r ≥ 0.996 and *p* < 0.001). In contrast, the PowerTap power meter registered a significantly higher (*p* < 0.05) percentage of time at <0.75 and >7.50 W·kg^−1^ and power profile at 1, 5 and 10 s. In conclusion, the data obtained in competitions by the two power meters were interchangeable. Nevertheless, the Power2Max power meter underestimated the pedaling power during short and high-intensity intervals (≤10.0 s and >7.50 W·kg^−1^) compared to the PowerTap power meter. Therefore, the analysis of these efforts should be treated with caution.

## 1. Introduction

The first registry of pedaling power output in cycle ergometer was dated in 1896, but the first portable power meters were not designed until the end of the 1980s (i.e., SRM, Balboa Instrument PowerPacer and Look Max One) [1,2]. Since then, these devices have been used to monitor training, to perform field-based performance tests, to analyze cycling competitions, and to evaluate changes in bicycle equipment [3].

Various portable power meters are available nowadays and can be classified according to their location on the bike (i.e., rear hub, crank, chainring, pedal, shoe, or handlebar) or to the sensor technology used (i.e., strain gauges, accelerometers, or multi-sensors to measure wind-speed, slope, etc.). More specifically, since the SRM patent expiration in 2007 (chainring power meter with strain gauges) some power meters with similar characteristics have been available (e.g., PowerTap C1, Quarq, Power2Max, FSA Powerbox). While Quarq’s validity and reliability has been questioned [4], the Power2Max power meter seems to be valid and reliable during submaximal pedaling (between 180–360 W) with the cyclist in seated position [3]. However, the PowerTap C1 and FSA Powerbox power meters have not been tested yet.

The Power2Max power meter was presented in the Eurobike 2010, and is now used by professional road cycling teams, triathletes, and amateur cyclists (www.power2max.de (accessed on 15 September 2022)). Nevertheless, two recent studies have questioned the interchangeability of the registry of different portable power meters in field conditions [3,5]. Consequently, Maier et al. [3] observed that the power output registered by different power meters is highly variable (even when they have been designed by the same manufacturer) and recommend further studies in field conditions with changes in ambient temperature, vibrations, or gear shifts. Shute et al. [6] observed that environmental temperature affected the registry of various power meters. Furthermore, Bouillod et al. [5] demonstrated that vibration and field conditions affect the power output measured.

This latter could condition the analysis and interpretation of both exercise intensity zones and power output profile of the cyclists [2,5,7], and their critical power [8,9]. These variables are widely used to quantify the competition load and to plan training [2]. However, to the best of our knowledge, no study has compared the influence of the power meter on these types of analysis during competition, possibly due to the mass added by each power meter or to the conflict of interest between sponsors (i.e., normally each cycling team uses only one power meter brand). Therefore, the main purpose of the present case study was to compare the interchangeability of the data obtained from two power meters (Power2Max vs. PowerTap) during road cycling competition.

## 2. Materials and Methods

A national-level under-23 male competitive cyclist (age: 21 years, height: 1.74 m, body mass: 64 kg; VO_2max_: 74.0 mL·kg^−1^·min^−1^; maximal aerobic power: 406 W; cycling experience: 13 years; typical training volume: 15,000 km per year) voluntarily participated and signed a written consent. The study was approved by the University Ethics Committee and met the requirements of the Declaration of Helsinki for research on human beings.

### 2.1. Procedures

The cyclist completed six road-cycling official competitions (five road races and one individual time-trial) of the elite under-23 regional and national calendar, between March and May of the same year (Table 1). During all of them, power output was simultaneously registered with two power meters (i.e., PowerTap and Power2Max) installed on the same road-racing bicycle (Scott Addict 30, Givisiez, Switzerland). The Power2Max meter, which registers power thanks to four strain gauges that measure torque and a cadence sensor (Power2Max Type S, Waldhufen, Germany), was fitted in the crankset and synchronized with a power control (Garmin Edge 705, Lenexa, KA, USA). On the other hand, the PowerTap meter registers power by using four strain gauges that measure torque and a speed sensor that measures the speed of the hub (PowerTap G3, Madison, WI, USA). This power meter was installed in the rear hub and synchronized with another power control (Garmin Edge 500, Lenexa, KA, USA). The two power controls were configured at 1 Hz sample frequency and installed on the handlebar stem. To avoid the influence of temperature on the calibration procedure [10], the bike remained for at least 30 min in the same ambient conditions in which the registry was obtained. Afterwards, the power meters were zeroed before performing each registry, according to the indications of the manufacturer, and a warm-up of 15 min at 150 W was standardized before starting the competition.

The six competitions were registered in both power meters from the beginning to the end of each stage. Power output measurements of both devices were analyzed with the same cycling performance software (Golden Cheetah 3.1, www.goldencheetah.org (accessed on 30 September 2022)). The identification of the exercise intensity zones and the power output profiles were obtained according to previous studies [11]. The first variable was the percentage of time with respect to the overall competition duration that the cyclist spent in each intensity zone (i.e., eleven intensities from <0.75 to >7.50 W·kg^−1^, with increments of 0.75 W·kg^−1^ between them). The second variable was the highest mean power that the cyclist held for a given period (i.e., twelve periods from 1 s to 60 min). Additionally, critical power, which was defined as the power asymptote of the hyperbolic relationship between power output and time to exhaustion [9], was also obtained from the power output profile, as previous studies did [6]. Finally, Normalized power, defined as the power output the cyclist could sustain if intensity were maintained constant without any variability [12].

### 2.2. Statistical Analysis

The results are expressed as mean ± SD. The SPSS+ version 20.0 statistical software was used (SPSS, Inc., Chicago, IL, USA). Spearman’s test was used to calculate the correlation coefficients between the two power meters, and the Wilcoxon signed-rank test to establish the statistical differences between means. Values of *p* < 0.05 were considered statistically significant.

## 3. Results

Table 1 shows the average power output, Normalized power and the cadence obtained in both power meters during the six competitions. The average power output (3.56 ± 0.68 and 3.62 ± 0.74 W·kg^−1^, *p* < 0.05), Normalized power (4.05 ± 0.45 and 4.11 ± 0.50 W·kg^−1^, *p* < 0.05), and cadence (95.7 ± 4.1 and 98.3 ± 3.5 rpm, *p* < 0.05) were lower in the PowerTap power meter than in the Power2Max power meter (ranges of the differences between 0.00–0.17 W·kg^−1^, 0.01–0.17 W·kg^−1^, and 2–4 rpm, respectively).

**Figure 1:**
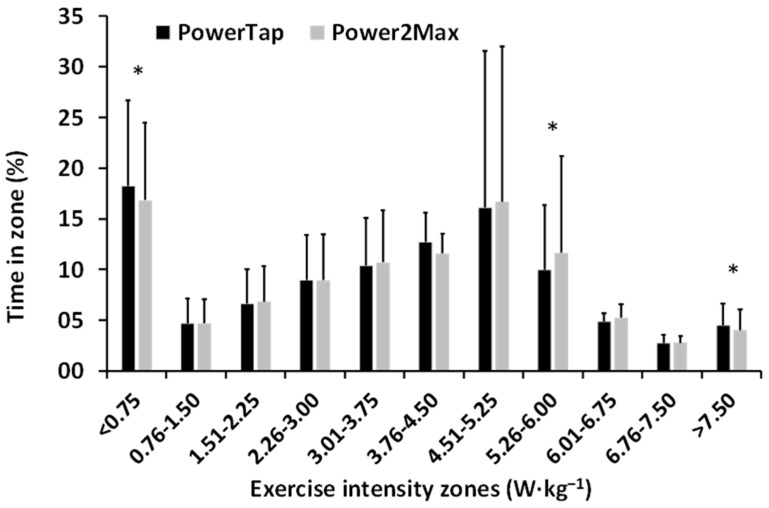
Mean ± SD of the time spent in each exercise intensity zone during the six official cycling competitions registered by the PowerTap and Power2Max power meters. * = significant differences (*p* < 0.05) between both power meters.

Figure 1 shows that the percentage of time at the different intensity zones was very similar between the PowerTap and the Power2Max power meters. Small differences were found at three intensity zones (PowerTap power meter values were higher at <0.75 and >7.50 W·kg^−1^, and Power2Max power meter values were higher at 5.26–6.00 W·kg^−1^). The spearman correlation test showed positive strong correlations between the two power meters in all zones during the six competitions (r = 0.986, *p* < 0.001).

**Figure 2:**
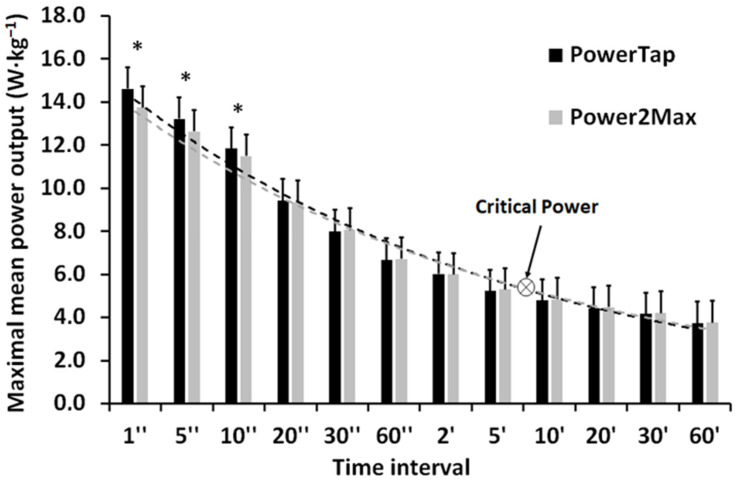
Mean ± SD of the maximal mean power output registered by the PowerTap and Power2Max power meters in each time interval during the six official cycling competitions. Critical power obtained from the PowerTap (dashed black line) and Power2Max registry (dashed grey line). * = significant differences (*p* < 0.05) between both power meters.

Figure 2 shows that the power profile registered by the PowerTap meter was significantly higher (*p* < 0.05) than in Power2Max meter at the time intervals of 1, 5, and 10 s (6.6, 4.9, and 2.8%, respectively), without differences in the rest of the intervals (between −1.0 and 1.3%). The Spearman correlation test showed positive correlations between the two power meters in all the intervals during the six competitions (r = 0.998, *p* < 0.001). The critical power obtained from both devices was very similar (5.06 and 5.11 W·kg^−1^, respectively).

## 4. Discussion

The main finding of this study was that the power output data for the PowerTap and Power2Max power meters were interchangeable when they were registered during road-cycling competitions. Values for average power and Normalized power (Table 1), time in power zones (Figure 1), power profile, and critical power (Figure 2) were very similar when comparing both power meters, with very high correlations between the two. However, the Power2Max power meter slightly overestimated average, Normalized, and critical power, and underestimated the pedaling power output during short and high-intensity intervals (i.e., 1–10 s and >7.50 W·kg^−1^). This is very important when analyzing efforts during training and competition, so future studies need to make an in-depth evaluation of it.

The small differences in both average and critical power (1–2%) could be explained by the location of the power meters (i.e., chainring vs. rear hub), because this power was dissipated in the deformation of the bike and chain friction, as previously stated by other authors [13,14]. They were similar to those described in studies that compared SRM and PowerTap meters [15], as well as to those observed on the development of a mathematical model of road cycling power [14]. Nevertheless, the differences at high pedaling power (2.8–6.6%), the highest cadence registered by the Power2Max power meter (1–4%, Table 1) and the possible influence of the weather (i.e., the average power was similar in two cloudy and/or rainy days, Table 1) justify the need for a study on the Power2Max meter’s validity, as previous studies about PowerTap and SRM power meters did [10,15]. In contrast, the Power2Max power meter could be used in studies where submaximal or incremental pedaling exercises are performed [16], considering its slight 1–2% overestimation of mean power output when compared to other cycle-ergometers [17,18,19].

According to Bertucci et al. [15], the PowerTap power meter slightly underestimates the pedaling power at high intensities with respect to the SRM meter, which should be added to the Power2Max power meter’s underestimation with respect to the PowerTap power meter found in the present study. During road cycling competition, power output values of ≥10 W·kg^−1^ have been registered during ≤30 s intervals for male professional cyclists [20,21,22]. Furthermore, it was demonstrated that the ability to repeat these high-intensity efforts was the difference between elite and non-elite male and female cyclists [7,22,23]. Therefore, it is very important to take into account the type of power meter for the registry and analysis of these efforts [24].

The main limitation of the present study was the participation of only one cyclist instead of several. As commented previously, this design was selected due to the difficulty of using two power meters during competition (i.e., mass added to the bike and conflict of interest between sponsors) and was similar to that used in previous studies on this subject [5,15].

## 5. Conclusions

The results from this case study suggest that the data for average power, time in power zones, power profile and critical power obtained from the Power Tap and Power2Max power meters during road cycling competitions might be interchangeable. Nevertheless, during short and high-intensity effort (≤10.0 s and >7.50 W·kg^−1^), the Power2Max power meter underestimates the pedaling power (2.8–6.6%). Therefore, this last registry should be treated with caution. However, further studies with a larger number of participants should confirm these findings.

## Figures and Tables

**Table 1:** Characteristics of the six official cycling competitions, average power output and cadence obtained by both the PowerTap and Power2Max power meters.

Competition	Distance (km)	Duration (hh:mm:ss)	Elevation gain (m)	Temperature (°C)	Weather	Average Power (W·kg^−1^)		Normalized Power (W·kg^−1^)		Average Cadence (rpm)	
						PT	P2M	PT	P2M	PT	P2M
RR 1	114.5	02:58:44	1145	18.8	Cloudy	3.18	3.18	3.72	3.75	93	96
RR 2	90	02:03:15	877	26	Sunny	3.36	3.42	4.00	4.08	97	101
RR 3	125	02:57:28	1096	15.4	Cloudy	3.40	3.48	3.98	4.08	96	98
RR 4	154	03:31:36	668	25.4	Sunny	3.10	3.15	3.73	3.77	92	96
RR 5	134.5	03:38:44	855	15.1	Cloudy and rainy	3.36	3.36	3.92	3.91	93	95
ITT 1	21.5	00:31:03	152	26	Sunny	4.93	5.10	4.92	5.09	103	104

RR = Road Race; ITT = Individual Time Trial; PT = Powertap; P2M = Power2Max.

## Data Availability

Data are contained within the article.
